# Antibodies against chemokine receptors CXCR3 and CXCR4 predict progressive deterioration of lung function in patients with systemic sclerosis

**DOI:** 10.1186/s13075-018-1545-8

**Published:** 2018-03-22

**Authors:** Florian Weigold, Jeannine Günther, Moritz Pfeiffenberger, Otavio Cabral-Marques, Elise Siegert, Duska Dragun, Aurélie Philippe, Ann-Katrin Regensburger, Andreas Recke, Xinhua Yu, Frank Petersen, Rusan Catar, Robert Biesen, Falk Hiepe, Gerd R. Burmester, Harald Heidecke, Gabriela Riemekasten

**Affiliations:** 1 Department of Rheumatology and Clinical Immunology, Charité University Hospital, Berlin, Germany; 2 Cell Autoimmunity Group, German Rheumatism Research Center (DRFZ), Berlin, Germany; 3 Department of Rheumatology, University of Lübeck, Lübeck, Germany; 4 Department of Nephrology and Critical Care Medicine, Charité University Hospital, Campus Virchow, Berlin, Germany; 5 Berlin Institute of Health, Berlin, Germany; 6 Department of Dermatology, University of Lübeck, Lübeck, Germany; 7 Research Center Borstel, Airway Research Center North (ARCN), Members of the German Center for Lung Research (DZL), Borstel, Germany; 8 CellTrend GmbH, Luckenwalde, Brandenburg Germany

**Keywords:** Anti-CXCR3, Anti-CXCR4, Systemic sclerosis, Interstitial lung disease, GPCR

## Abstract

**Background:**

The chemokine receptors CXCR3 and CXCR4 are involved in the pathogenesis of fibrosis, a key feature of systemic sclerosis (SSc). It is hypothesized that immunoglobulin (Ig)G antibodies (abs) against these two receptors are present in patients with SSc and are associated with clinical findings.

**Methods:**

Anti-CXCR3 and anti-CXCR4 ab levels were measured in 449 sera from 327 SSc patients and in 234 sera from healthy donors (HD) by enzyme-linked immunosorbent assay (ELISA). In SSc, ab levels were compared with clinical data in a cross-sectional and longitudinal setting. Protein expression of CXCR3 and CXCR4 on peripheral blood mononuclear cells (PBMCs) was analyzed in 17 SSc patients and 8 HD by flow cytometry.

**Results:**

Anti-CXCR3 and anti-CXCR4 ab levels were different among SSc subgroups compared with HD and were highest in diffuse SSc patients. The ab levels strongly correlated with each other (*r* = 0.85). Patients with SSc-related interstitial lung disease (SSc-ILD) exhibited higher ab levels which negatively correlated with lung function parameters (e.g., *r* = −0.5 and *r* = −0.43 for predicted vital capacity, respectively). However, patients with deterioration of lung function showed lower anti-CXCR3/4 ab levels compared with those with stable disease. Frequencies and median fluorescence intensities (MFI) of CXCR3^+^ and CXCR4^+^ PBMCs were lower in SSc patients compared with HD and correlated with the severity of skin and lung fibrosis. They correlated with the severity of skin and lung fibrosis.

**Conclusions:**

Anti-CXCR3/4 abs and their corresponding receptors are linked with the severity of SSc-ILD. Antibody levels discriminate patients with stable or decreasing lung function and could be used for risk stratification.

**Electronic supplementary material:**

The online version of this article (10.1186/s13075-018-1545-8) contains supplementary material, which is available to authorized users.

## Background

Systemic sclerosis (SSc) is a severe autoimmune disease characterized by vasculopathy and fibrosis. In this disease, SSc-related interstitial lung disease (SSc-ILD) is one of the most frequent causes of mortality [1] due to activation and accumulation of T cells, neutrophils, and monocytes in the inflammation sites. C-X-C motif chemokine receptor (CXCR)3 and CXCR4 are G protein-coupled receptors (GPCRs) mediating the migration of different cells including lymphocytes, endothelial progenitor cells, and stem cells [2, 3]. CXCR3, which can bind to several proinflammatory chemokines, is induced on naive T cells upon activation by interferon gamma-inducible ligands present in inflamed tissues. As a result, CXCR3^+^ T cells, predominantly Th1 cells, are recruited to sites of inflammation [4]. CXCR3 is also expressed on plasmacytoid dendritic and natural killer (NK) cells [4, 5]. An increased number of CXCR3^+^ cells were also found in lupus nephritis and in atherosclerotic lesions secondary to systemic lupus erythematosus (SLE) [6, 7]. CXCR4 is a specific receptor for stromal-derived factor 1 (SDF-1, also called C-X-C motif chemokine ligand 12 or CXCL12), a molecule with potent chemotactic activity for lymphocytes and neutrophils [5]. Involvement of the CXCR4/SDF-1 axis is discussed in several diseases including inflammatory bowel diseases, ischemia/reperfusion injury, and in idiopathic pulmonary fibrosis. CXCR4^+^ cells as well as their ligands CXCL11 and CXCL12 have been shown to be associated with the pathogenesis of pulmonary complications in patients with autoimmune diseases [8–11]. Furthermore, CXCL11 levels in bronchoalveolar lavage fluid (BALF) from SSc patients predicted progressive SSc-ILD [12].

As recently reported by our group, immunoglobulin (Ig)G from SSc patients is able to induce ILD and vasculopathy in C57BL/6 mice indicating a pathogenic role of antibodies. IgG from SSc patients directly enhances *in vitro* migration of T cells and neutrophils correlating with anti-angiotensin II type I receptor (AT1R)/endothelin receptor type A (ETAR) antibody (ab) levels [13, 14]. In addition, anti-AT1R and anti-ETAR abs induced the production of different cytokines, Ca^2+^, and chemokines by various cells, which were inhibited by AT1R or ETAR blockers [13–18]. However, the inhibition was partially incomplete or even absent indicating antibody-mediated activation of other receptors. In SSc patients, anti-AT1R and anti-ETAR ab levels were predictive for vascular complications such as pulmonary arterial hypertension (PAH) or digital ulcers [15–17, 19], but they were not able to predict deterioration of pulmonary fibrosis. Assuming the existence of antibodies against CXCR3 and CXCR4, our group has developed enzyme-linked immunosorbent assays (ELISAs) to measure their concentrations in sera. Here, we asked whether anti-CXCR3 and anti-CXCR4 antibodies or the receptors could be used as biomarkers for SSc or for SSc-related organ damage.

## Methods

We analyzed 449 sera from 327 consecutive SSc patients from the Charité University Hospital for anti-CXCR3 abs; 425 sera from 312 SSc patients were analyzed for the presence of anti-CXCR4 abs. Epidemiologic data of the 327 SSc patients are shown in Additional file 1: Table S1 and baseline lung function parameters at the time point of anti-CXCR3/4 ab detection are given in Additional file 1: Table S2. The mean time between disease onset and determination of ab values is 8.5 ± 6.9 years for anti-CXCR3 and 8.4 ± 6.9 years for anti-CXCR4.

In comparison, sera from 234 healthy donors (HD) (68.4% male and 31.6% female, with a mean age of 36.6 ±12.8 years) were analyzed.

SSc patients were divided into different disease subsets according to the criteria of the EUSTAR and DNSS network [20–25]. Comorbidities were assessed in routine clinical practice as documented in the medical charts. Only those comorbidities that were present in at least 10 patients were analyzed. If multiple ab samples/clinical assessments were registered per patient, only the latest sample was considered in cross-sectional analyses. SSc patients with a follow-up period of at least 3 years were analyzed to identify the predictive capacity of the ab.

SSc-ILD was defined according to the guidelines from the German Network of Systemic Sclerosis and the definition has been applied and used for several studies. Accordingly, diagnosis of SSc-ILD was established when bilateral basal fibrosis occurred, confirmed by chest x-ray and/or high-resolution computed tomography (HR-CT) scan together with restrictive pulmonary abnormalities on pulmonary function tests (forced vital capacity (FVC) < 80%). Regularly, in patients with limited cutaneous disease and particularly in those with anti-Centromer ab and normal lung function, an HR-CT scan is not required and the x-ray is sufficient for primary diagnosis. Lung fibrosis was only quantified indirectly by lung function tests; quantitative analyses of HR-CT scans were not performed in our clinical routine setting.

Here, in our cross-sectional analyses for anti-CXCR3 ab, 35% of the patients have a diagnosed SSc-ILD, 23% of them with FVC < 70%. In analyses for anti-CXCR4 ab, 33% of the patients have a diagnosed SSc-ILD, 31% of them with FVC < 70%.

### Detection of anti-CXCR3 and anti-CXCR4 IgG by solid-phase assay

Anti-CXCR3 and anti-CXCR4 IgG ab were measured by a commercially available sandwich ELISA (CellTrend GmbH Luckenwalde, Germany). Briefly, duplicate samples of a 1:100 serum dilution were incubated at 4 °C for 2 h. All sera were analyzed by personnel blinded to the clinical characteristics of the patients.

#### Isolation of peripheral blood mononuclear cells and flow cytometry

CXCR3 and CXCR4 protein expression was measured on peripheral blood mononuclear cell (PBMC) subpopulations from 17 SSc patients (eight diffuse cutaneous (d)SSc patients, 8 SSc patients with limited cutaneous (l)SSc, and one patient with overlap syndrome) and eight HD by flow cytometry. Epidemiologic data of the patients are given in Additional file 1: Table S1. PBMCs were isolated using Ficoll-Paque gradient centrifugation as described previously [18], identified by their corresponding CD surface antigens (CD4, T helper cells; CD8, cytotoxic T cells; CD19, B cells; CD14, monocytes), stained ex vivo or immediately after isolation, and fixed with 4% paraformaldehyde. Cytometric assays were performed using an LSR II flow cytometer (BD Biosciences) and analyzed with FlowJo software (TreeStar, Ashland, OR, USA). Frequencies were normalized to the corresponding isotype control, calculating the difference between isotype control (between 0 and 5%) and receptor-stained cells within the identical gate. For median of fluorescence intensity (MFI), the ratio of the MFI of receptor-stained cells and the MFI of isotype control was calculated. The other ab, as well as methods describing T-cell isolation and chemotaxis assays, are given in Additional file 1: Supplemental Material and Methods.

### Statistical analysis

Associations between categorical data were measured using Chi squared or Fisher’s exact test. Nonparametric Kruskal-Wallis test with Dunn-Bonferroni post-hoc tests, Mann-Whitney *U* test and Spearman’s rho were used to examine noncategorical, non-normal distributed values. Receiver operating characteristic (ROC) analyses were performed to identify the best cut-off points for ab levels for patients with and without deterioration of lung function. Log-rank test and Cox proportional hazards model were performed to identify the predictive value of anti-CXCR3 and anti-CXCR4 ab regarding deterioration of lung function parameters (estimation of hazard ratios and baseline adjustment of lung function parameters, age, and sex).

For receptor expression, statistics were performed using Prism5 software (GraphPad Inc., La Jolla, USA). A value of *p* ≤ 0.05 was interpreted as statistically significant.

## Results

### Anti-CXCR3 and anti-CXCR4 antibody levels show strong correlations with each other and are different among the different disease subsets

**Fig. 1:**
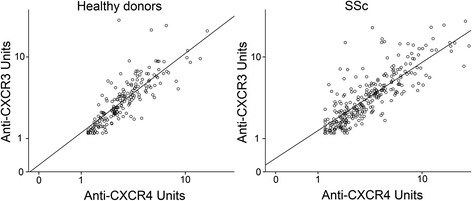
Anti-CXCR3 and anti-CXCR4 antibody levels were strongly correlated in healthy donors (left) as well as in systemic sclerosis (SSc) patients (right)

Anti-CXCR3 and anti-CXCR4 ab levels in sera from SSc patients were compared with those from HD (Additional file 1: Figure S1a, b). Median values of anti-CXCR3 abs were 3.5 Units (U) (95% confidence interval (CI) 2.9–3.8) in HD and 3.8 U in SSc patients (95% CI 3.2–4.2), showing no significant differences (*p* = 0.12). On the other hand, median values of anti-CXCR4 abs were higher in SSc patients (3.3 U, 95% CI 3–3.8) compared with HD (2.7 U, 95% CI 2.5–3; *p* ≤ 0.001). However, 12.2% and 17.2% of the SSc patients displayed anti-CXCR3 and anti-CXCR4 ab levels above the 95th percentile in comparison to HD, respectively. The levels of anti-CXCR3 and anti-CXCR4 abs strongly correlate with each other in HD and in SSc patients (*r* = 0.89 and *r* = 0.85, respectively; *p* ≤ 0.001, *n* = 198 and *n* = 312; Fig. 1). In SSc patients, anti-CXCR3 and anti-CXCR4 ab levels were weakly and negatively correlated with the corresponding ages at assessment (*r* = −0.2, *p* ≤ 0.001; and *r* = −0.2, *p* ≤ 0.001, respectively) and with the age at SSc diagnosis (*r* = −0.24, *p* ≤ 0.001; and *r* = −0.27, *p* ≤ 0.001, respectively), but not with disease duration (data not shown). In contrast, HD show positive correlations for both anti-CXCR3 and anti-CXCR4 ab levels with age (*r* = 0.13, *p* ≤ 0.05; and *r* = 0.16, p ≤ 0.05, respectively).

**Fig. 2:**
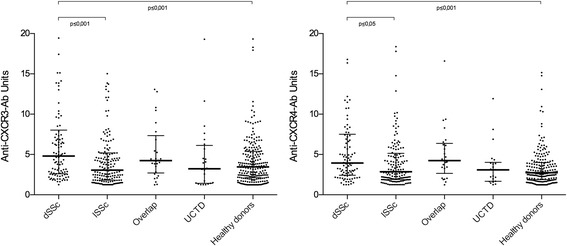
Anti-CXCR3 (left) and anti-CXCR4 (right) antibody levels in diffuse cutaneous systemic sclerosis (dSSc), in patients with limited cutaneous systemic sclerosis (lSSc), with overlap syndromes (Overlap), with undifferentiated connective tissue disease (UCTD), and in healthy controls. *P* values with significant differences are shown

Anti-CXCR3 ab levels were higher in patients with diffuse cutaneous systemic sclerosis (dSSc) compared with patients with limited cutaneous systemic sclerosis (lSSc) (*p* ≤ 0.001) and HD (*p* ≤ 0.001; Fig. 2); 17.6% of the dSSc patients revealed anti-CXCR3 ab levels above the 95th percentile. Similarly, anti-CXCR4 ab levels were higher in dSSc patients compared with those in lSSc patients (*p* ≤ 0.05) and HD (*p* ≤ 0.001). In line with this, anti-topoisomerase I ab-positive patients have slightly higher anti-CXCR3 levels than anti-topoisomerase I ab-negative patients (median 4.1 vs. 4.7 U; *p* ≤ 0.05, *n* = 252). No significant differences were observed for anti-CXCR4 abs. Patients with overlap syndromes revealed markedly increased ab levels compared with HD (*p* = 0.056); 25.5% of the dSSc patients have anti-CXCR4 ab levels above the 95th percentile of the HD.

### Anti-CXCR3 and anti-CXCR4 antibodies are markers for lung involvement

The cross-sectional analyses showed that the subgroup of patients with confirmed SSc-ILD and FVC < 70% had significantly lower median anti-CXCR3 and anti-CXCR4 ab levels than those with FVC ≥ 70% (2.9 U vs. 4.8 U, *p* ≤ 0.01, *n* = 82; and 2.8 U vs. 5.0 U, *p* ≤ 0.05, *n* = 78, respectively). In the next step, we studied correlations between ab levels and different metric clinical parameters of SSc patients. Significant negative correlations exist between anti-CXCR3 ab levels and the predicted percentages of vital capacity (VC), FVC, total lung capacity (TLC) and the diffusing capacity for carbon monoxide measured by a single breath (DLCO-SB). Anti-CXCR4 abs revealed similar correlations with lung function parameters (Table 1), indicating that high anti-CXCR3 and anti-CXCR4 ab levels are associated with more severely impaired lung function. In line with this, higher median anti-CXCR3 and anti-CXCR4 ab levels were present in patients with SSc-ILD compared with those without ILD (3.4 U vs. 4.1 U, *p* ≤ 0.05; and 3.1 U vs. 3.9 U, *p* ≤ 0.05, respectively).

**Table 1:** Correlations between anti-CXCR3 and anti-CXCR4 antibody levels and lung function parameters in systemic sclerosis patients

Lung function parameter	Anti-CXCR3			Anti-CXCR4		
	SRT-r	*p* values	*n*	SRT-r	*p* values	*n*
VC (%)	–0.50	≤ 0.001	71	−0.43	≤ 0.001	58
FVC (%)	−0.27	≤ 0.001	284	−0.2	≤ 0.001	271
TLC (%)	−0.25	≤ 0.001	253	−0.21	≤ 0.001	240
DLCO-SB (%)	−0.32	≤ 0.01	274	−0.26	≤ 0.05	64
DLCO/VA (%)	−0.20	= 0.08	245	−0.16	= 0.20	64
FEV1 (%)	–0.18	≤ 0.01	278	−0.17	≤ 0.01	265
FEV1/VC (%)	+0.25	≤ 0.05	71	+0.2	= 0.13	58

*R* values of Spearman’s rho (SRT-r) as well as *p* values are shown

Vital capacity (VC), forced vital capacity (FVC), total lung capacity (TLC), the diffusing capacity for carbon monoxide (DLCO) measured by a single breath (DLCO-SB), DLCO/VA, forced expiratory volume in 1 s (FEV1), and Tiffeneau index (FEV1/VC) are given

No correlations were present between anti-CXCR3 and anti-CXCR4 ab levels and modified Rodnan Skin Score (mRSS), mean pulmonary arterial pressure (mPAP), systolic pulmonary arterial pressure (sPAP), or left ventricular ejection fraction (LVEF) (data not shown). For anti-CXCR3 ab levels, weak correlations existed with erythrocyte sedimentation rate (ESR) levels (*r* = 0.16, *p* ≤ 0.05, *n* = 251), but not with C-reactive protein (CRP) values (data not shown).

Anti-CXCR3 and anti-CXCR4 antibodies were also associated with other clinical symptoms as shown in Additional file 1: Tables S3 and S4.

### High anti-CXCR3/4 antibody levels correspond to and predict stable lung function

SSc patients with 3-year follow-up investigations were analyzed to identify the role of anti-CXCR3/4 abs to predict progression in SSc-ILD. SSc patients were grouped into those having > 10% deterioration in DCLO-SB, DLCO/VA, FVC, forced expiratory volume in 1 s (FEV1), and TLC values, and those with stable lung function parameters. Ab samples were obtained at the beginning of the 3-year follow-up. ROC analyses (Youden’s-Index) were performed for all lung function parameters to identify the best cut-off values which can discriminate between stable and progressive diseases within 3 years. For clinical practicality, the median value (6.2 U for anti-CXCR3 and 5.18 U for anti-CXCR4) is used for all parameters.

In general, patients with lower levels of anti-CXCR3/CXCR4 abs have progressively impaired lung function during this follow-up period (Table 2).

**Table 2:** Higher anti-CXCR3 and anti-CXCR4 antibody levels are associated with stable lung function parameters

Parameter	Patients above cut-off (%)		Patients below cut-off (%)		*p* values	Number of analyzed patients	Hazard ratios
	Deterioration ≥ 10%	No deterioration	Deterioration ≥ 10%	No deterioration			
Anti-CXCR3 antibodies							
FVC (%)	25.0	75.0	59.3	40.7	≤ 0.01	59	0.32
FEV1 (%)	23.5	76.5	71.1	28.9	≤ 0.001	72	0.22
DLCO-SB (%)	36.4	63.6	63.6	36.4	≤ 0.05	55	0.47
DLCO/VA (%)	42.4	57.6	70.4	29.6	≤ 0.05	60	0.38
Anti-CXCR4 antibodies							
FVC (%)	22.2	77.8	70.0	30.0	≤ 0.001	47	0.2
FEV1 (%)	33.3	66.7	78.6	21.4	≤ 0.001	61	0.27
TLC (%)	33.3	66.7	64.7	35.3	≤ 0.05	44	0.33
DLCO-SB (%)	43.3	56.7	75.0	25.0	≤ 0.05	46	0.46
DLCO/VA (%)	39.4	60.6	82.4	17.6	≤ 0.001	50	0.24

Patients with antibody levels above and below cut-off, with and without deterioration in forced vital capacity (FVC), forced expiratory volume in 1 s (FEV1), total lung capacity (TLC), the diffusing capacity for carbon monoxide (DLCO) measured by a single breath (DLCO-SB), and DLCO/VA within 3 years, *p* values (log-rank test) as well as number of analyzed patients are shown

Hazard ratios derived from the Cox proportional hazards model highlight the relationship between deterioration in lung function parameters and anti-CXCR3/anti-CXCR4 antibody levels

**Fig. 3:**
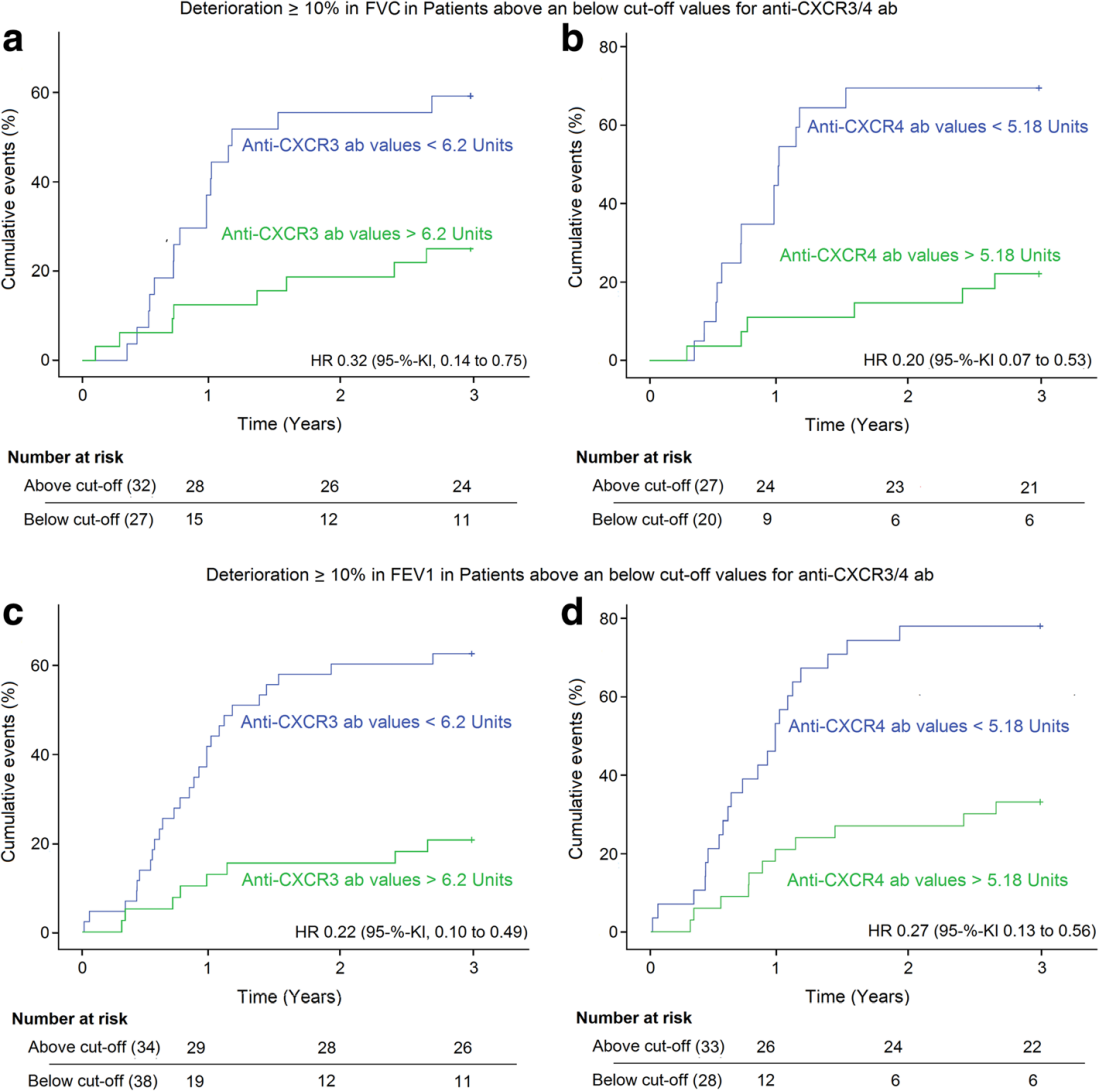
Cumulative events indicate the capacity of anti-CXCR3 (**a,c**) and anti-CXCR4 (**b,d**) antibody (ab) levels to predict deterioration in forced vital capacity (FVC) (**a,b**) and forced expiratory volume in 1 s (FEV1) (**c,d**) by ≥ 10% within 3 years of follow-up. *P* values (log-rank test) are given in Table 2. HR hazard ratio, 95-%-KI 95% confidence interval

Cumulative percentages of patients with deterioration > 10% of predicted FVC and FEV1 levels are shown in Fig. 3.

Within 12 months, 44% of the patients with anti-CXCR3 ab levels < 6.2 U showed a reduction in FVC values ≥ 10%, but this was only true for 13% of the patients with high ab levels. Similar results were obtained at month 24 (56% versus 19%) and at month 36 (59% versus 25%). Baseline adjustment for lung function parameters, age, and sex did not reveal significant differences in the FVC deterioration. Anti-CXCR4 ab levels appear to discriminate better between patients with and without FVC deterioration. At month 12, 55% of the SSc patients with anti-CXCR4 ab levels < 5.18 U revealed deterioration in FVC values, but this was the case in only 11% of the patients with higher ab levels. Similar results were obtained at months 24 (70% versus 15%) and at months 36 (70% versus 22%). Low anti-CXCR3 and anti-CXCR4 ab levels were also predictive for deterioration in TLC, DLCO-SB, and DLCO/VA values.

### CXCR3 and CXCR4 protein expressions are related to signs of fibrosis

The protein expressions of CXCR3 and CXCR4 were analyzed in PBMCs of SSc patients as well as HD. The percentages of peripheral blood monocytes, CD8^+^ T cells, and B cells expressing CXCR3 as well as CXCR4 were lower in SSc patients compared with HD (Additional file 1: Figure S2 shows the percentages of cells expressing CXCR3**)**. In addition, MFIs were reduced for CXCR3 and CXCR4 in monocytes, CD8^+^ T cells, and B cells of SSc patients compared with those found in HD (Additional file 1: Figure S2).

**Fig. 4:**
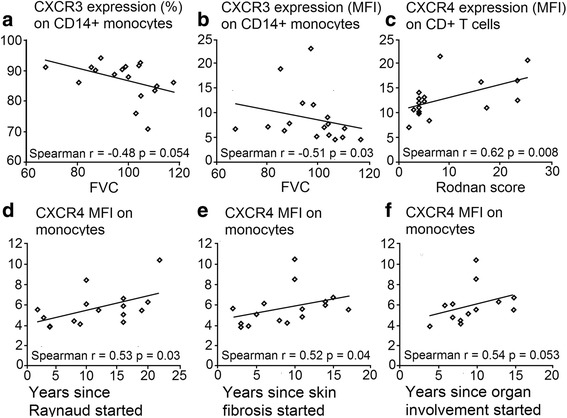
Frequency of CXCR3-positive CD14^+^ monocytes among CD14^+^ monocytes (**a**) as well as CXCR3 density (**b**) on CD14^+^ monocytes show a negative correlation with the predicted percentages of forced vital capacity (FVC). (**c**) CXCR4 density on CD4^+^ T cells related to isotype controls correlated with modified Rodnan Skin Score (mRSS). Correlation between CXCR4 expressions on monocytes (MFIs) with years since diagnosis (**d**), start of fibrosis (**e**), and since first organ involvement (**f**, first non-Raynaud symptom). Statistical analyses were performed by Spearman’s correlation; *p ≤ 0.05. MFI median fluorescence intensity

In CD14^+^ monocytes, both the percentage of CXCR3-expressing cells as well as their MFI inversely correlated with the FVC values (Fig. 4a, b). The MFI of CXCR4-positive CD4^+^ T cells correlated well with the mRSS (Fig. 4c). The MFI of CXCR4 expression on monocytes correlated with the years since onset of Raynaud’s phenomenon (*r* = 0.53, *p* ≤ 0.05), with years since the start of fibrosis (*r* = 0.52, *p* ≤ 0.05), and with years since the first organ involvement (*r* = 0.54, *p* ≤ 0.05) (Fig. 4f).

## Discussion

In the present study, autoantibodies against the chemokine receptors CXCR3 and CXCR4 were mainly linked to the presence of SSc-ILD. Patients with dSSc and those with lung fibrosis revealed higher anti-CXCR3 and anti-CXCR4 ab levels compared with those with lSSc and without SSc-ILD. The ab levels weakly and negatively correlated with lung function parameters. In addition, CXCR3 and CXCR4 receptor expression in PBMCs correlated with the extent of lung and skin fibrosis. However, the deterioration of lung function parameters was associated with low anti-CXCR3 and anti-CXCR4 ab levels. In addition, low anti-CXCR3 and anti-CXCR4 ab levels predicted progressive impairment of lung function parameters.

The involvement of CXCR3 and CXCR4 has already been discussed in the pathogenesis of systemic sclerosis. Cipriani et al. have shown an upregulation of CXCR4 and SDF-1 in the skin of early systemic sclerosis patients [26, 27]. However, increased, normal, and decreased percentages of CXCR3^+^ cells have been described in SSc [28–30]. Here, percentages of CXCR3 and CXCR4^+^ cytotoxic T cells, B cells, and monocytes were lower in SSc patients compared with HD. This could reflect reduced activation of cells, downregulation of receptor expression by chronic stimulation, or migration of these cells from the blood stream into the tissue; this remains to be studied.

Here, anti-CXCR3 and anti-CXCR4 ab levels are shown to be very good markers of predicting lung fibrosis and are, therefore, candidates to stratify patients for disease progression, e.g., in clinical studies. This is the main finding of our study. The association of high ab levels with signs of lung fibrosis and reduced progression of lung fibrosis seems to be a contradiction at first glance. A similar observation was found for regulatory T cells (Treg), which strongly correlate with disease activity in lupus [31]. This could be suggestive for their pathogenic role. Nevertheless, enrichment of Treg improved lupus activity illustrating that high Treg numbers do not necessarily indicate a pathogenic role of the Treg [31]. Similar to the beneficial role of Treg, the prediction of progressive deterioration of FVC values by low anti-CXCR3/4 levels suggests a protective effect of the antibodies, which needs to be further evaluated.

In our previous studies, anti-CXCR3/4 levels and anti-AT1R/ETAR ab levels were not able to predict progressive SSc-ILD (data not shown). Anti-CXCR3 and anti-CXCR4 ab levels correlate moderately with those of the anti-AT1R and anti-ETAR ab (*r* = 0.39 to *r* = 0.43, data not shown). Functionally, anti-AT1R/ETAR abs are stimulatory and agonistic to their natural ligands [32]. The previously described correlation between cell migration and the levels of anti-AT1R and anti-ETAR abs indicate a dominant role of anti-AT1R/ETAR abs in cell migration in SSc [14, 15]. As shown in the supplementary results (Additional file 1: Figure S3), IgG fractions from patients with high anti-CXCR3/4 abs also mediate migration via CXCR3 and probably via CXCR4 as suggested by blocking experiments with specific receptor blockers. Migration of lymphocytes was not dependent on the anti-CXCR3/4 ab levels (data not shown). Sequence identity between CXCR3 and CXCR4 abs and between anti-CXCR4 and anti-AT1R abs has been described [33, 34]. According to this, anti-CXCR3/4 ab levels as well as relative high receptor expressions could interfere with the effects of anti-AT1R/ETAR abs on cell migration. However, alterations in the anti-CXCR3/4 ab levels as well as in CXCR3 and CXCR4 expression were linked with fibrosis, suggesting a disturbed CXCR3/4 system in this condition.

Our study has some limitations. The assessment was performed in a routine clinical setting leading to some missing clinical parameters. Based on the earlier availability of the CXCR3 ELISA, anti-CXCR4 abs were not measured in all patients. However, in the vast majority, anti-CXCR3 and anti-CXCR4 abs were measured in parallel in one individual. Also, we did not study patients with other diseases. This is a limitation that should be addressed in the future as well as analyzing cross-reactions of the antibodies. In addition, we used lung function parameters as surrogate markers for lung fibrosis and did not measure lung fibrosis by quantitative CT scans. In this study, we did not investigate the predictive value of these antibodies on mortality, nor did we investigate the effects of disease-modifying agents on the results. Finally, specific functions of the abs remain to be studied.

## Conclusions

In conclusion, we have identified novel anti-GPCR abs against the chemokine receptors CXCR3 and CXCR4 in patients with systemic sclerosis linking autoimmunity with interstitial lung disease. Antibody levels discriminate patients with stable or decreasing lung function and could be used for risk stratification. We suggest a functional role of anti-CXCR3/4 abs and highlight their potential contribution to the orchestra of other antibodies.

## Additional file

Additional file 1: Supplementary material. (ZIP 236 kb)

## Abbreviations

ab: Antibody
AT1R: Anti-angiotensin II type I receptor
BALF: Bronchoalveolar lavage fluid
CI: Confidence interval
CRP: C-reactive protein
CXCR: C-X-C motif chemokine receptor
DLCO-SB: Diffusing capacity for carbon monoxide by a single breath
dSSc: Diffuse cutaneous systemic sclerosis
ELISA: Enzyme-linked immunosorbent assay
ESR: Erythrocyte sedimentation rate
ETAR: Endothelin receptor type A
FEV1: Forced expiratory volume in one second
FVC: Forced vital capacity
GPCR: G protein-coupled receptor
HD: Healthy donors
HR-CT: High-resolution computed tomography
Ig: Immunoglobulin
ILD: Interstitial lung disease
lSSc: Limited cutaneous systemic sclerosis
LVEF: Left ventricular ejection fraction
MFI: Median fluorescence intensity
mPAP: Mean pulmonary arterial pressure
mRSS: Modified Rodnan Skin Score
PAH: Pulmonary arterial hypertension
PBMC: Peripheral blood mononuclear cell
ROC: Receiver operating characteristic
SDF-1: Stromal-derived factor 1
SLE: Systemic lupus erythematosus
sPAP: Systolic pulmonary arterial pressure
SSc: Systemic sclerosis
TLC: Total lung capacity
Treg: Regulatory T cells
VC: Vital capacity

## References

[1] Tyndall AJ, Bannert B, Vonk M, Airo P, Cozzi F, Carreira PE (2010). Causes and risk factors for death in systemic sclerosis: a study from the EULAR Scleroderma Trials and Research (EUSTAR) database. Ann Rheum Dis.

[2] Groom JR, Luster AD (2011). CXCR3 ligands: redundant, collaborative and antagonistic functions. Immunol Cell Biol.

[3] Murdoch C (2000). CXCR4: chemokine receptor extraordinaire. Immunol Rev.

[4] Lacotte S, Brun S, Muller S, Dumortier H. CXCR3, inflammation, and autoimmune diseases. Ann N Y Acad Sci. 2009;1173:310–7. 10.1111/j.1749-6632.2009.04813.x 19758167

[5] Hummel S, Van Aken H, Zarbock A (2014). Inhibitors of CXC chemokine receptor type 4: putative therapeutic approaches in inflammatory diseases. Curr Opin Hematol.

[6] Enghard P, Humrich JY, Rudolph B, Rosenberger S, Biesen R, Kuhn A (2009). CXCR3+CD4+ T cells are enriched in inflamed kidneys and urine and provide a new biomarker for acute nephritis flares in systemic lupus erythematosus patients. Arthritis Rheum.

[7] Clement M, Charles N, Escoubet B, Guedj K, Chauveheid MP, Caligiuri G (2015). CD4+CXCR3+ T cells and plasmacytoid dendritic cells drive accelerated atherosclerosis associated with systemic lupus erythematosus. J Autoimmun.

[8] Tourkina E, Bonner M, Oates J, Hofbauer A, Richard M, Znoyko S (2011). Altered monocyte and fibrocyte phenotype and function in scleroderma interstitial lung disease: reversal by caveolin-1 scaffolding domain peptide. Fibrogenesis Tissue Repair.

[9] Andersson-Sjoland A, de Alba CG, Nihlberg K, Becerril C, Ramirez R, Pardo A (2008). Fibrocytes are a potential source of lung fibroblasts in idiopathic pulmonary fibrosis. Int J Biochem Cell Biol.

[10] Costello CM, McCullagh B, Howell K, Sands M, Belperio JA, Keane MP (2012). A role for the CXCL12 receptor, CXCR7, in the pathogenesis of human pulmonary vascular disease. Eur Respir J.

[11] Antoniou KM, Soufla G, Lymbouridou R, Economidou F, Lasithiotaki I, Manousakis M (2010). Expression analysis of angiogenic growth factors and biological axis CXCL12/CXCR4 axis in idiopathic pulmonary fibrosis. Connect Tissue Res.

[12] Sfriso P, Cozzi F, Oliviero F, Caso F, Cardarelli S, Facco M (2012). CXCL11 in bronchoalveolar lavage fluid and pulmonary function decline in systemic sclerosis. Clin Exp Rheumatol.

[13] Günther J, Kill A, Becker MO, Heidecke H, Rademacher J, Siegert E (2014). Angiotensin receptor type 1 and endothelin receptor type A on immune cells mediate migration and the expression of IL-8 and CCL18 when stimulated by autoantibodies from systemic sclerosis patients. Arthritis Res Ther.

[14] Kill A, Tabeling C, Undeutsch R, Kühl AA, Günther J, Radic M (2014). Autoantibodies to angiotensin and endothelin receptors in systemic sclerosis induce cellular and systemic events associated with disease pathogenesis. Arthritis Res Ther.

[15] Becker MO, Kill A, Kutsche M, Guenther J, Rose A, Tabeling C (2014). Vascular receptor autoantibodies in pulmonary arterial hypertension associated with systemic sclerosis. Am J Respir Crit Care Med.

[16] Günther J, Rademacher J, van Laar JM, Siegert E, Riemekasten G (2015). Functional autoantibodies in systemic sclerosis. Semin Immunopathol.

[17] Riemekasten G, Philippe A, Näther M, Slowinski T, Müller DN, Heidecke H (2011). Involvement of functional autoantibodies against vascular receptors in systemic sclerosis. Ann Rheum Dis.

[18] Rademacher J, Kill A, Mattat K, Dragun D, Siegert E, Günther J (2016). Monocytic angiotensin and endothelin receptor imbalance modulate secretion of the profibrotic chemokine ligand 18. J Rheumatol.

[19] Avouac J, Riemekasten G, Meune C, Ruiz B, Kahan A, Allanore Y (2015). Autoantibodies against endothelin 1 type A receptor are strong predictors of digital ulcers in systemic sclerosis. J Rheumatol.

[20] Walker UA, Tyndall A, Czirjak L, Denton C, Farge-Bancel D, Kowal-Bielecka O (2007). Clinical risk assessment of organ manifestations in systemic sclerosis: a report from the EULAR Scleroderma Trials and Research group. Ann Rheum Dis.

[21] Hunzelmann N, Genth E, Krieg T, Lehmacher W, Melchers I, Meurer M (2008). The registry of the German network for systemic scleroderma: frequency of disease subsets and patterns of organ involvement. Rheumatology.

[22] Carwile LeRoy E, Black C, Fleischmajer R, Jablonska S, Krieg T, Medsger TA (1988). Scleroderma (systemic sclerosis): classification, subsets and pathogenesis. J Rheumatol.

[23] Bennett RM (1990). Scleroderma overlap syndromes. Rheum Dis Clin N Am.

[24] Rodnan GP, Fennell RH (1962). Progressive systemic sclerosis sine scleroderma. JAMA.

[25] Mosca M, Neri R, Bombardieri S (1999). Undifferentiated connective tissue diseases (UCTD): a review of the literature and a proposal for preliminary classification criteria. Clin Exp Rheumatol.

[26] Cipriani P, Franca Milia A, Liakouli V, Pacini A, Manetti M, Marrelli A (2006). Differential expression of stromal cell-derived factor 1 and its receptor CXCR4 in the skin and endothelial cells of systemic sclerosis patients: pathogenetic implications. Arthritis Rheum.

[27] Makino H, Aono Y, Azuma M, Kishi M, Yokota Y, Kinoshita K (2013). Antifibrotic effects of CXCR4 antagonist in bleomycin-induced pulmonary fibrosis in mice. J Med Investig.

[28] Jiang D, Liang J, Hodge J, Lu B, Zhu Z, Yu S (2004). Regulation of pulmonary fibrosis by chemokine receptor CXCR3. J Clin Invest.

[29] Fujii H, Hasegawa M, Takehara K, Mukaida N, Sato S (2002). Abnormal expression of intracellular cytokines and chemokine receptors in peripheral blood T lymphocytes from patients with systemic sclerosis. Clin Exp Immunol.

[30] Shimizu S, Yoshinouchi T, Niimi T, Ohtsuki Y, Fujita J, Maeda H (2007). Differing distributions of CXCR3- and CCR4-positive cells among types of interstitial pneumonia associated with collagen vascular diseases. Virchows Arch.

[31] Humrich JY, Riemekasten G (2017). Low-dose IL-2 therapy—a complex scenario that remains to be further explored. Nat Rev Rheumatol.

[32] Cabral-Marques O, Riemekasten G (2016). Vascular hypothesis revisited: role of stimulating antibodies against angiotensin and endothelin receptors in the pathogenesis of systemic sclerosis. Autoimmun Rev.

[33] Schmidt D, Bernat V, Brox R, Tschammer N, Kolb P (2015). Identifying modulators of CXC receptors 3 and 4 with tailored selectivity using multi-target docking. ACS Chem Biol.

[34] Andrews SP, Cox RJ (2016). Small Molecule CXCR3 Antagonists. J Med Chem.

