# Supplementary material for: Antibodies against chemokine receptors CXCR3 and CXCR4 predict progressive deterioration of lung function in patients with systemic sclerosis
Source: Arthritis Res Ther. 2018 Mar 22;20:52. doi: 10.1186/s13075-018-1545-8 (PMC5863842; doi:10.1186/s13075-018-1545-8)
Supplement: Supplementary file 1 — Supplementary material. (ZIP 236 kb) [file 13075_2018_1545_MOESM1_ESM.zip › SUPPLEMENTS.docx]

**Supplementary Material and Methods**

**Antibodies used for flow cytometry**

Mouse IgG anti-human CD3 cyanin 5 (clone UCHT-1) and mouse IgG anti-human CD14 Alexa 700 (clone TM1) were obtained from the German Rheumatism Research Center (DRFZ, Berlin, Germany). Mouse IgG anti-human CD4 allophycocyanin-cyanin 7 (clone RPA-T4, cat. No. 300518), mouse IgG anti-human CD183 (CXCR3) Alexa 488 (clone G025H7, cat. no. 353710) and the respective isotype control mouse IgG1 Alexa 488 (clone MOPC-21, cat. no. 400129), mouse IgG anti-human CD184 (CXCR4) phycoerythrin (clone 12G5, cat. no. 306506) and the respective isotype control mouse IgG2a phycoerythrin (MOPC-173, cat. no. 400212) were obtained from Biolegend (San Diego, CA, USA).

**T cell isolation and chemotaxis assays**

T cells were isolated by depletion of non-T cells performing magnetic activated cell sorting with the Pan T cell kit (Miltenyi, Bergisch Gladbach, Germany). Purity of the selected populations was usually more than 95% as assessed by flow cytometry.

T cells were cultured overnight at a concentration of 1-2 million/mL in RPMI1640 medium with Glutamax, 1% IgG-free FCS, Penicillin 100 U/mL and Streptomycin100 µg/mL (all PAA Laboratories, Austria) in a humidified atmosphere with 5% CO2 at 37°C. Next day cells were seeded at a concentration of 1.0-1.3 million cells/mL in anti-CD3/CD28 coated well plates (10 and 5µg/mL, respectively, coated overnight at 4°C) and cultured for three days in the presence of IL-2 (10µg/mL) under the conditions described above. Cells were washed in PBS/BSA and resuspended in assay medium (RPMI1640 with 0.5% BSA). Chemotaxis experiments were performed in transwell cell culture chambers with a polycarbonate membrane of 5µm pore size (Costar Corning Incorporated, USA). 106 cells in 100µL were placed to the inserts. The inserts were positioned into individual wells containing 600µL assay medium with or without indicated IgG at a concentration of 100µg/mL. Receptor antagonists AMG 487 for CXCR3 (N-1-[(3-4(-Ethoxyphenyl)-3,4-dihydro-4-oxopyrido[2,3-d]pyrimidin-2-yl]ethyl]-N-(3-pyridinylmethyl)-4-(trifluoromethoxy) ben-zeneacetamide) and AMD 3100 for CXCR4 ([1-[4-(1,4,8,11-Tetrazacyclotetradec- 1-ylmethyl)phenyl] methyl]-1,4,8,11-tetrazacyclo-tetradecan) were added to both the inserts and the corresponding wells at a concentration of 100 ng/µl. Appropriate concentrations of the antagonists and the IgG were determined in experiments using CXCL11 and CXCL12, the ligands for CXCR3 and CXCR4, respectively. Plates were incubated for 2 hours in a humidified atmosphere with 5% CO2 at 37°C. The number of cells which were migrated into the well was determined with a Neubauer hemocytometer (Marienfeld, Germany).

**Suppl. Fig. 1** Anti-CXCR3 (a) and anti-CXCR4 ab levels (b) in healthy donors (HD) versus SSc patients.

[FigSup1.png]

**Suppl. Fig. 2** Percentages of cells expression CXCR3 and median fluorescence intensity of cells expression CXCR4 (CD 184) in monocytes, CD8+ T cells, and in B cells in Healthy donors (HD) versus SSc patients. P values (Mann-Whitney U-Test).

[FigSup2.png]

**Suppl. Fig. 3** Chemotaxis of isolated T cells is induced by the natural ligands CXCL11 and CXCL12 as well as by SSc IgG (SKL 13, 206, 497 = IgG Patient) and higher compared to IgG from healthy donors (NC 17). a) Ligand-induced migration is reduced by CXCR3 (AMD3100) and CXCR4 receptor antagonists (AMG 487). b) Chemotaxis by SSc IgG (Patient) is significantly reduced by the CXCR3 antagonist and markedly reduced by the CXCR4 antagonist. Statistical analyses were performed by Mann-Whitney U-Test (SSc IgG vs. SSc IgG plus receptor blockers), *p<0.05

[FigSup3.png]

**Suppl. Tab. 1:** Epidemiologic data of SSc patients analysed for anti-CXCR3/4 antibodies (SSc patients for ab) as well as for CXCR3/4 expression (SSc cohort expression).

| Patient‘s data | SSc patients for ab | lcSSc | dSSc | Overlap | UCTD | SSc cohort expression |
| --- | --- | --- | --- | --- | --- | --- |
| Female (%) | 284 (86,9) | 150 (90,4) | 79 (77,5) | 26 (89,7) | 23 (95,8) | 15 (88.2) |
| Male (%) | 43 (13,1) | 16 (9,6) | 23 (22,5) | 3 (10,3) | 1 (4,2) | 2 (11.8) |
| All (%) | 327 (100) | 166 (100) | 102 (100) | 29 (100) | 24 (100) | 17 (100) |
| Age in years (SD) | 56.46 (14.71) | 59.57 (14.71) | 53.29 (14.05) | 50.59 (14.78) | 56.42 (11.91) | 55 (11.4) |
| Age at diagnosis in years (SD) | 48.43 (15.75) | 50.48 (14.55) | 45.25 (14.92) | 44.29 (14.21) | 51.69 (12.73) | 48.4 (10.4) |

**Suppl. Tab. 2:** Baseline lung function parameters of SSc-Patients

| Parameter | Anti-CXCR3 ab analysis  Mean ± std. deviation, (n) | Anti-CXCR4 ab analysis  Mean ± std. deviation, (n) |
| --- | --- | --- |
| VC (%) | 88.3 ±16.0 (71) | 89.8 ±16.4 (58) |
| FVC (%) | 92.7 ± 18.6 (284) | 93.2 ± 18.7 (271) |
| TLC (%) | 98.2 ± 17.5 (253) | 98.6 ± 17.6 (240) |
| DLCO-SB (%) | 63.7 ± 17.1 (274) | 64.0 ± 17.1 (261) |
| DLCO/VA (%) | 77.4 ± 15.9 (245) | 77.7 ± 15.9 (231) |
| FEV1 (%) | 93.6 ± 17.4 (253) | 93.1 ± 17.5 (265) |
| FEV1/VC (%) | 104.9 ± 11.2 (71) | 104.9 ± 11.2 (58) |

**Suppl. Tab. 3:** Associations of SSc-Patients with CXCR3/4 ab level above 95. percentile of healthy donors (HD) with clinical symptoms.

| Parameter | Prevalence | Patients with ab level  > 95. percentile of HD | | P values | Number of analysed patients |
| --- | --- | --- | --- | --- | --- |
|  |  | with manifestation | w/o manifestation |  |  |
| Anti-CXCR3 antibodies | | | | | |
| Joint manifestations | 190 (58.1%) | 29 (15,3%) | 11 (8.0%) | ≤ 0.05 | 327 |
| Osteoarthritis | 26 (9.5%) | 0 (0%) | 26 (13%) | ≤ 0.05 | 273 |
| Anti-CXCR4 antibodies | | | | | |
| Cardiac arrhythmias | 20 (7.6%) | 7 (35%) | 36 (14.8%) | ≤ 0.05 | 263 |
| Cardiac failure | 10 (3.8%) | 4 (40%) | 39 (15.4%) | ≤ 0.05 | 263 |
| Cardiovascular diseases | 140 (53.2%) | 30 (21,4) | 13 (10,6%) | ≤ 0.05 | 263 |

**Suppl. Tab. 4:** Median CXCR3/4 ab levels of SSc-Patients with and without clinical symptoms.

| Parameter | Median ab level with manifestation (U) | Median ab level w/o manifestation (U) | P values | Number of analysed patients |
| --- | --- | --- | --- | --- |
| Anti-CXCR3 antibodies | | | | |
| Muscular weakness | 4.24 | 3.37 | ≤ 0.05 | 321 |
| Osteoarthritis | 2.38 | 3.80 | ≤ 0.05 | 273 |
| Proteinuria | 1,83 | 3,85 | ≤ 0.01 | 321 |
| Sec. Sjoegren’s syndrome | 2.76 | 3.80 | = 0.06 | 273 |
| Fibromyalgia | 2.2 | 3.76 | = 0.06 | 273 |
| Anti-CXCR4 antibodies | | | | |
| Muscle weakness | 3.43 | 3.12 | ≤ 0.05 | 306 |
| Muscle pain | 4.82 | 3.11 | ≤ 0.05 | 187 |
| Sec. Sjoegren’s syndrome | 2.25 | 3.69 | ≤ 0.05 | 263 |
