# Supplementary figures and images for: Antibodies against chemokine receptors CXCR3 and CXCR4 predict progressive deterioration of lung function in patients with systemic sclerosis
Source: Arthritis Res Ther. 2018 Mar 22;20:52. doi: 10.1186/s13075-018-1545-8 (PMC5863842; doi:10.1186/s13075-018-1545-8)

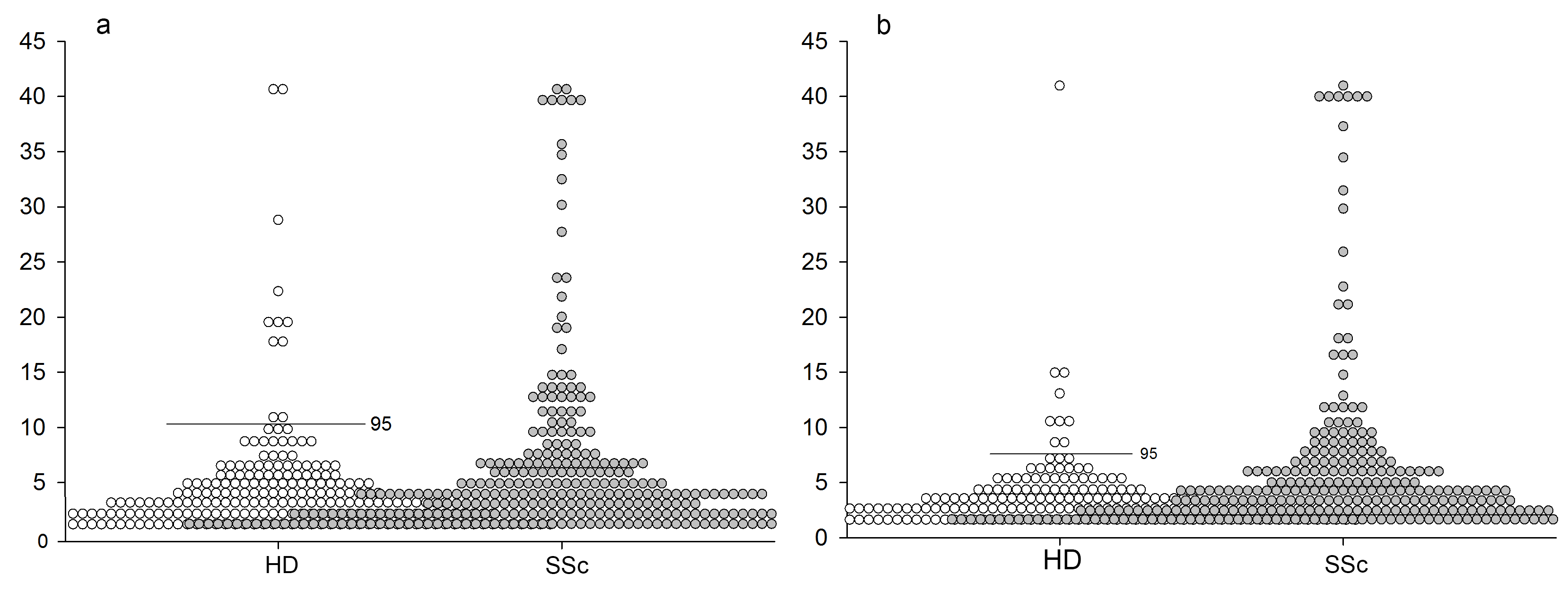

Supplement: Supplementary file 1 — Supplementary material. (ZIP 236 kb) [file 13075_2018_1545_MOESM1_ESM.zip › FigSup1.png]

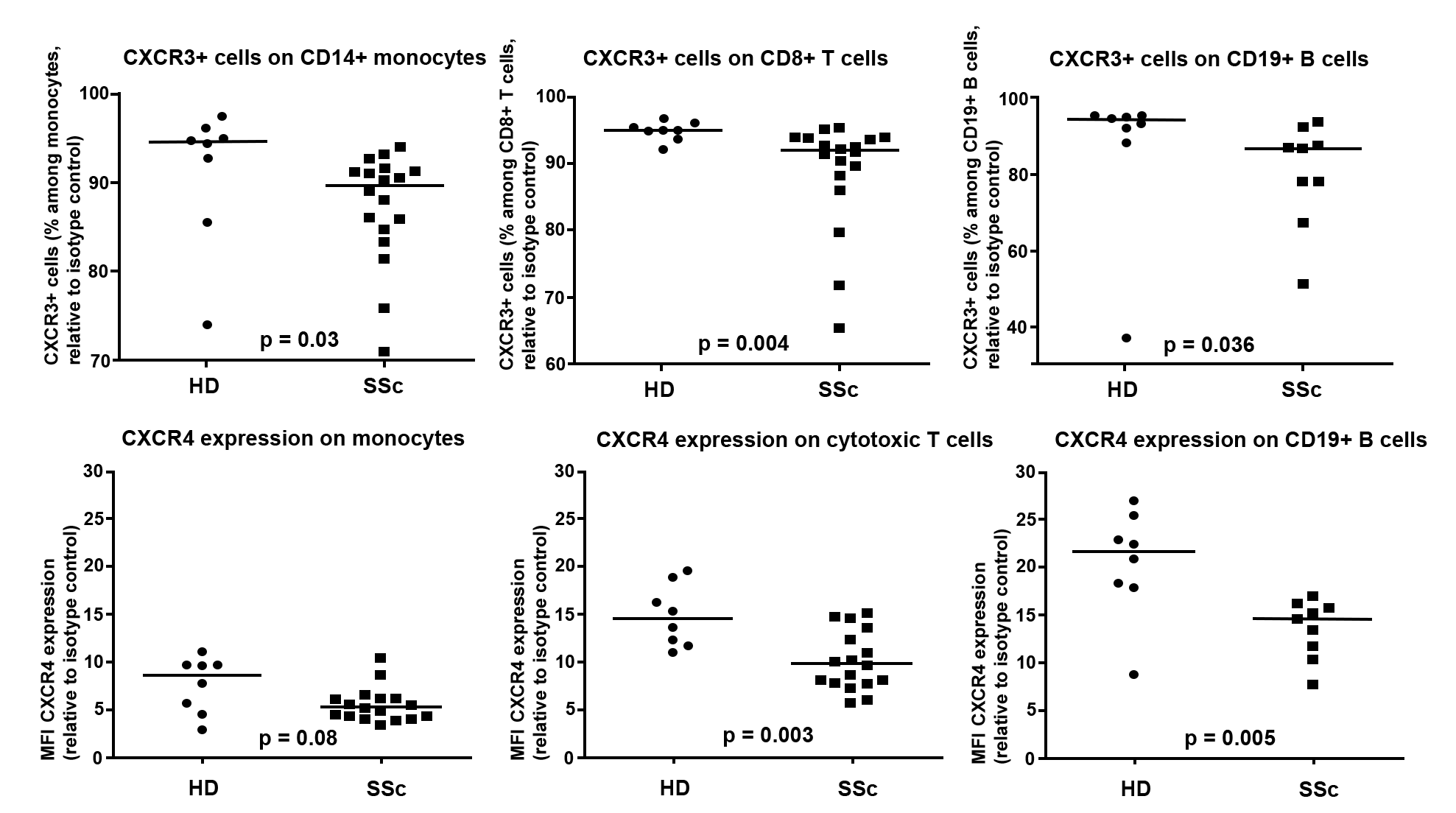

Supplement: Supplementary file 1 — Supplementary material. (ZIP 236 kb) [file 13075_2018_1545_MOESM1_ESM.zip › FigSup2.png]

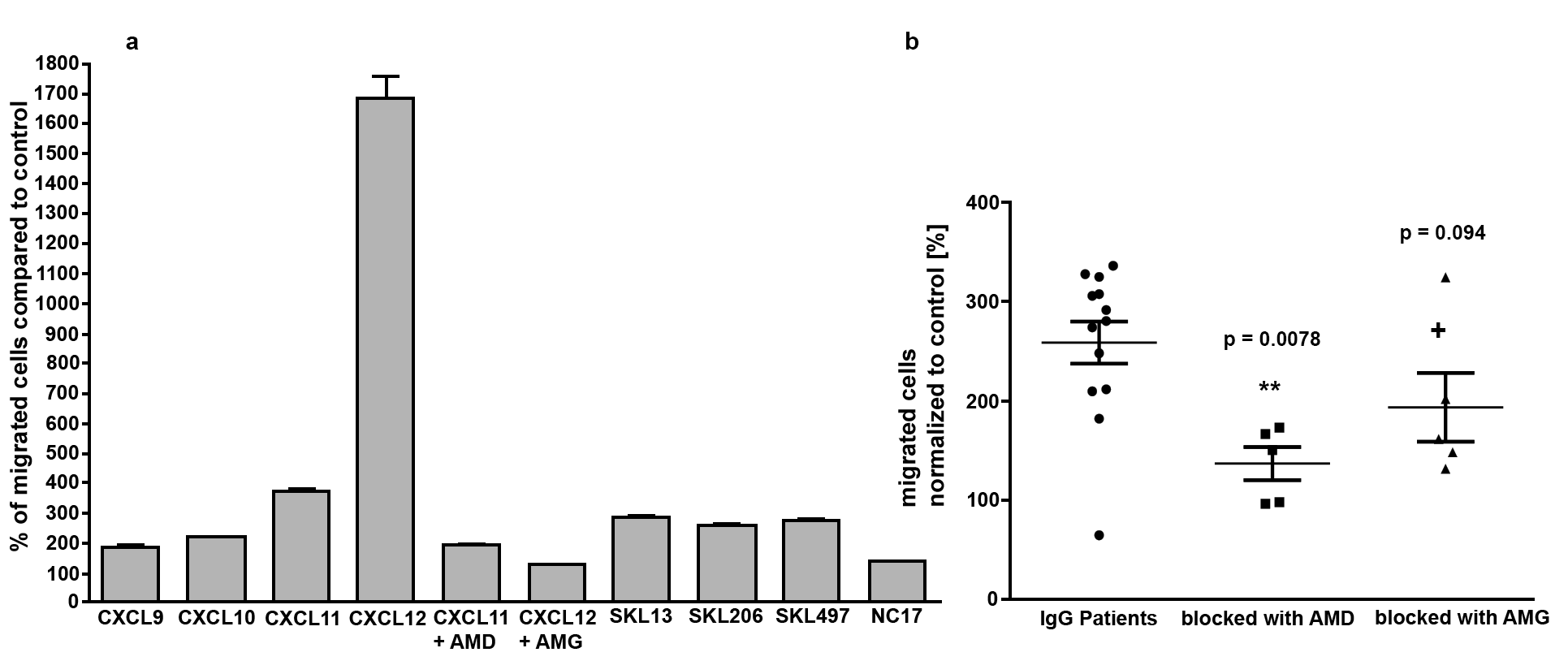

Supplement: Supplementary file 1 — Supplementary material. (ZIP 236 kb) [file 13075_2018_1545_MOESM1_ESM.zip › FigSup3.png]
